# Recent Advances in Single-Particle Electron Microscopic Analysis of Autophagy Degradation Machinery

**DOI:** 10.3390/ijms21218051

**Published:** 2020-10-28

**Authors:** Yiu Wing Sunny Cheung, Sung-Eun Nam, Calvin K. Yip

**Affiliations:** Life Sciences Institute, Department of Biochemistry and Molecular Biology, The University of British Columbia, Vancouver, BC V6T 1Z3, Canada; sywcheun@student.ubc.ca (Y.W.S.C.); snama@mail.ubc.ca (S.-E.N.)

**Keywords:** autophagy, selective autophagy, Atg proteins, single-particle electron microscopy, cryo-EM

## Abstract

Macroautophagy (also known as autophagy) is a major pathway for selective degradation of misfolded/aggregated proteins and damaged organelles and non-selective degradation of cytoplasmic constituents for the generation of power during nutrient deprivation. The multi-step degradation process, from sequestering cytoplasmic cargo into the double-membrane vesicle termed autophagosome to the delivery of the autophagosome to the lysosome or lytic vacuole for breakdown, is mediated by the core autophagy machinery composed of multiple Atg proteins, as well as the divergent sequence family of selective autophagy receptors. Single-particle electron microscopy (EM) is a molecular imaging approach that has become an increasingly important tool in the structural characterization of proteins and macromolecular complexes. This article summarizes the contributions single-particle EM have made in advancing our understanding of the core autophagy machinery and selective autophagy receptors. We also discuss current technical challenges and roadblocks, as well as look into the future of single-particle EM in autophagy research.

## 1. Introduction

Conserved from yeast to humans, macroautophagy (hereafter autophagy) is a key degradation pathway that maintains cellular homeostasis through targeting cytoplasmic macromolecules and organelles to the lysosomes or lytic vacuoles for a breakdown. In multi-cellular organisms, autophagy plays a pivotal role in development and differentiation by allowing cells to change its protein composition in response to external cues [1,2]. Autophagy also participates in self-defense by targeting intracellular microbes, including bacteria, viruses, and protozoa, for degradation [3,4]. Dysregulation of autophagy has been linked to many human diseases ranging from neurodegeneration to different types of cancers [5,6]. A comprehensive mechanistic understanding of the autophagy pathway may, therefore, provide insights into the molecular etiologies of human diseases and reveal avenues for therapeutic intervention.

Autophagy degrades cytoplasmic material, both selectively and non-selectively. Selective autophagy operates at basal level in normal, nutrient abundant conditions and handles the removal of misfolded/aggregated proteins and damaged organelles [7,8]. On the other hand, non-selective autophagy is typically induced by stress conditions, such as nutrient deprivation, and promotes bulk degradation of proteins, lipids, and carbohydrates that allows the cell to generate energy to power essential processes [9,10,11]. For both forms of autophagy, cytoplasmic cargo is first sequestered into a double-membrane transport vesicle known as the autophagosome. The cargo-laden autophagosome is then delivered to and subsequently fuses with the lysosome (or the vacuole in yeast and plant cells), where its content is degraded by hydrolytic enzymes inside this lytic organelle. However, autophagosome biogenesis occurs de novo at the phagophore assembly site (PAS) in yeast cells (or the omegasome in mammalian cells) in non-selective autophagy, whereas this process is mediated by the cargo in selective autophagy [7,10,11]. 

The landmark discovery of the autophagy-related (*ATG*) genes in the 1990s by yeast genetic screening generated a framework for molecular investigation of the autophagy pathway [12]. Amongst this ensemble of genes, the 18 encoding Atg proteins that make up the “core autophagy machinery” have since become the major foci of autophagy research. These mostly conserved Atg proteins, which operate in the context of five functional modules (the Atg1 complex, the autophagy-specific phosphatidylinositol 3-kinase complex, the Atg8 conjugation system, the Atg12 conjugation system, and the integral membrane protein Atg9 and the Atg2-Atg18 complex), play essential roles in autophagy initiation and autophagosome biogenesis [9,13,14]. In addition to the core machinery, a sequence-divergent family of proteins known as selective autophagy receptors has gained attention in the field [15]. Identified in yeast and higher eukaryotes, these selective autophagy receptors serve as adaptors to link the cargo to the core autophagy machinery for its encapsulation and subsequent degradation. 

A key approach in characterizing the core autophagy machinery and selective autophagy receptors is through elucidating their molecular structures. X-ray crystallography and NMR spectroscopy have provided the initial thrusts in these investigations, and several excellent reviews have summarized their contributions in advancing our mechanistic understanding of autophagy [14,16,17,18,19,20,21]. Single-particle electron microscopy (EM) is an emerging method to obtain structural information of proteins and protein complexes. It utilizes the transmission electron microscope (TEM) to obtain 2-dimensional (2D) images of individual protein/protein complex “particles”, which would subsequently allow reconstruction of their 3-dimensional (3D) structures. Single-particle EM specimens can be prepared by negative staining, where a thin layer of sample is overlaid on the EM grid and then fixed and dried in a heavy metal salt solution that provided high contrast, due to the stronger scattering power of the large atoms in the solution. Although the procedure is simple, negatively stained specimens are subject to flattening and other potential staining artifacts, and the resulting images are relatively low resolution. The second method to prepare a single-particle EM specimen is vitrification, in which a very thin layer of sample on the EM grid is rapidly frozen so that the protein/protein complexes are embedded in a thin layer of amorphous ice. This technique allows the specimen to maintain in its near-native state and preserve high-resolution details, but the contrast between the specimen and the amorphous ice is low, due to the absence of the heavy atom stain, thus yielding images with high background noise. More recently, the development of direct electron detectors and new image processing algorithms have dramatically improved the resolution and data quality achievable by single-particle EM, in particular cryo-EM, and catapulted this imaging technology into mainstream structural biology research [22,23]. In this review, we will highlight recent achievements made by single-particle EM, both negative stain and cryo-EM, in the structural characterization of core autophagy machinery modules, selective autophagy receptors, and their cargoes. We will also describe ongoing challenges and look into the future of single-particle EM in autophagy research.

## 2. Structural Investigations of the Atg1/ULK Complex

The different modules of the core autophagy machinery operate in a hierarchical fashion to mediate autophagosome biogenesis at the phagophore assembly site [24,25]. At the top of this hierarchy are the Atg1 complex and the orthologous ULK complex in mammalian cells. The Atg1/ULK complex is a protein kinase complex that integrates signaling input from the nutrient and hormonal sensing TOR (target of rapamycin) signaling network to activate downstream factors to coordinate the initiation of non-selective autophagy. In nutrient-rich conditions, TOR complex 1 (TORC1), one of the two signaling hubs of the TOR pathway, inhibits autophagy by suppressing the activity of the Atg1/ULK complex [26,27]. During starvation, TORC1 inactivation results in the upregulation of Atg1/ULK complex activity and a corresponding increase in autophagic degradation [27,28,29]. Much of our structural knowledge on the Atg1/ULK complex has been derived from studies on the budding yeast *Saccharomyces cerevisiae* Atg1 complex. This complex is a hetero-pentameric assembly composed of the serine/threonine protein kinase Atg1, the regulatory subunits Atg13 and Atg17, and two accessory subunits Atg29 and Atg31 that bind constitutively to Atg17 [30,31,32]. Single-particle EM has generated data that enabled an integrated understanding of the overall architecture of this complex and by providing a versatile method to test hypotheses derived from structural investigations. 

**Figure 1:**
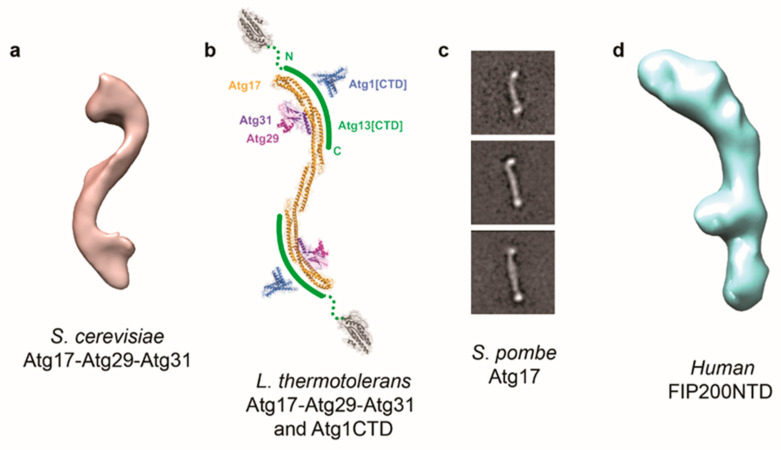
3D electron microscopy (EM) reconstructions and subunit arrangement of Atg1 complex and FIP200. (**a**) Negative stain EM reconstruction of *Saccharomyces cerevisiae* Atg17-Atg29-Atg31. (**b**) Proposed subunit arrangement of *Lachancea thermotolerans* Atg1 complex based on results from negative stain electron microscopy, X-ray crystallography, and cross-linking coupled to mass-spectrometry. (**c**) Representative 2D class averages of *Schizosaccharomyces pombe* Atg17 from negative stain EM. (**d**) 3D cryo-EM reconstruction of human FIP200NTD (EMD-21325).

The first application of single-particle EM on the *S. cerevisiae* Atg1 complex was a negative stain-based investigation on the subassembly composed of Atg17, Atg29, and Atg31 (hereafter Atg17-Atg29-Atg31) (Figure 1a) [33]. This study was reported after the Hurley group study, which used X-ray crystallography and small-angle X-ray scattering to determine the first structural data on the Atg17-Atg29-Atg31 core containing truncated Atg29 from thermophilic yeast *Lachancea thermotolerans* [34]. Notably, 2D averages and a 3D EM reconstruction from this study verified the overall S-shaped architecture of the *S. cerevisiae* Atg17-Atg29-Atg31 containing full-length subunits (Figure 1b) [33]. Despite at lower resolution, results from complementary EM-based labeling analysis confirmed the subunit organization of this subassembly, with dimeric Atg17 generating a central scaffold and two copies of the Atg29/Atg31 globular heterodimer attached to each of the two concave surfaces of the scaffold. The unique overall architecture of Atg17-Atg29-Atg31 led the Hurley group to propose an “early vesicle tethering” model. In this model, activation of the Atg1 complex leads to the displacement of Atg29-Atg31 from the concave surfaces of Atg17, which opens up sites for binding and subsequent tethering of Atg9-containing small vesicles. The tethering of Atg9 vesicles is believed to promote the formation of the phagophore [35,36], the precursor membrane that expands and sequesters cargo and eventually forms the autophagosome. The ability to directly visualize proteins without crystallization allows single-particle EM to test different aspects of this model. Negative stain EM analysis of *S. cerevisiae* Atg17 showed that the dimeric protein cannot stably adopt an S-shaped structure in the absence of Atg29 and Atg31 [33]. Further characterization of Atg17-Atg29-Atg31 containing phosphomimetic mutants of Atg29, the presumed “active” state of Atg17-Atg29-Atg31, showed that these mutations did not alter the location of Atg29-Atg31 along the Atg17 scaffold [37]. Collectively, these data suggested that the curvature of Atg17-Atg29-Atg31 may not necessarily be evolved for accommodating the high curvature of the small-sized Atg9 vesicles. Instead, the curvature-inducing functions of Atg29-Atg31 may serve to constrain the overall length of the Atg17 scaffold tethers Atg9 vesicles at an optimal distance. Interestingly, the estimated end-to-end length of Atg17 closely matches that of previously characterized multi-subunit tethering complexes that function in conventional membrane trafficking pathways [38,39,40,41]. 

Although different research groups have used X-ray crystallography to characterize the globular N-terminal domain of Atg13, C-terminal domain of Atg1 and to map the Atg17-binding regions of Atg13, the full heteropentameric *S. cerevisiae* Atg1 complex remained refractory to structural analysis, due, in part, to its sophisticated composition and the presence of intrinsically disordered regions on Atg1 and Atg13. Single-particle EM, in conjunction with chemical cross-linking coupled to mass spectrometry, enabled the characterization of the *S. cerevisiae* Atg1 “core” complex composed of full-length Atg17, Atg29, Atg31, the C-terminal domain of Atg1, and the C-terminal domain of Atg13 [42]. More specifically, results from negative stain EM-labeling analysis showed that both the Atg1 C-terminal domain and Atg13 C-terminal domain localize to the “tips” of the S-shaped scaffold formed by Atg17-Atg29-Atg31. Cross-linking coupled to mass spectrometry generated a set of distance restraints that derivates the structural model of the core yeast Atg1 complex incorporating high-resolution crystallographic structures of subunits and subassemblies (Figure 1b). An interesting aspect of this structural model is that the N-terminal kinase domain of Atg1 is projected away and do not make direct contacts with its regulators Atg17 and Atg13, suggesting that Atg13 and Atg17 modulate Atg1 in a manner distinct from classical kinase regulators. 

Like the yeast Atg1 complex, the mammalian ULK complex also contains three core components: The serine/threonine kinase and the Atg1 orthologue ULK1 or ULK2, ATG13, and FIP200, the scaffolding subunit that is four times larger than yeast Atg17. However, the ULK complex contains a unique subunit called ATG101 in place of Atg29 and Atg31 [43,44]. Subsequent large-scale genome sequence analysis revealed that the presence of Atg29/Atg31 and ATG101 is mutually exclusive for organisms with available genome sequences [45]. Biochemical and X-ray crystallography-based structural analysis showed that ATG101 binds and stabilizes the N-terminal HORMA domain of ATG13 [46], but the effect of the absence of Atg29/Atg31 on the ULK complex is unknown. This question was initially addressed using the model organism fission yeast *Schizosaccharomyces pombe*, which has an Atg101-containing Atg1 complex and lacks Atg29 and Atg31. Although the full *S. pombe* Atg1 could not be reconstituted, systematic protein-protein interaction analysis of the *S. pombe* Atg1 complex indicated that the absence of Atg29 and Atg31 did not affect the interactions between the three core subunits (Atg1, Atg13, Atg17) [47]. Interestingly, negative stain EM analysis of *S. pombe* Atg17 showed that it adopts an extended dimeric architecture that lacks inherent curvature (Figure 1c). In other words, at least for the *S. pombe* Atg1 complex, the absence of Atg29 and Atg31 did not result from the scaffolding subunit evolving an inherent curvature. 

More recently, the Hurley group made key breakthroughs in the structural investigation of the human ULK complex. They initially used X-ray crystallography to determine the high-resolution claw-shaped structure of the C-terminal region of FIP200 (residues 1458-1594) [48]. This was followed by two landmark studies on FIP200 that visualized first the N-terminal domain of FIP200 (FIP200 NTD) and subsequently full-length FIP200 [49,50]. In the first study, negative stain EM analysis showed that FIP200 NTD is inherently dimeric and adopts a broad range of conformations ranging from “bent rods” to “C-shape” [50]. However, the addition of ATG101 and ATG13 restricts this flexibility in a similar manner that Atg29-Atg31 has on yeast Atg17 as the FIP200 NTD-ATG101-ATG13 subassembly adopts mainly a C-shaped architecture [33,34,50]. These investigators were also able to visualize, by negative stain EM, single particles of a subassembly containing FIP200 NTD, ATG13, ATG101, and the C-terminal domain of ULK1 [51]. This observation, in conjunction with multiangle light scattering analysis, revealed an unexpected subunit stoichiometry with each dimeric FIP200 NTD-binding asymmetrically to one copy of ATG13, ATG101, and ULK1 [50]. They also succeeded in obtaining a ~9 Å cryo-EM map of FIP200 NTD in complex with the minimal binding region known as the ATG13 middle domain (ATG13 MR) (Figure 1d). Although the limited resolution precluded visualization of individual amino acid side chains, the cryo-EM map showed resemblance to the scaffold-like domain and ubiquitin-like domain of TBK1. In a follow-up study, these investigators developed a strategy to purify full-length FIP200, and this enabled them to use negative stain EM to visualize individual FIP200 particles. These images not only revealed the highly extended nature of FIP200 with end-to-end distances ranging from 320 to 940 Å, but also showed that the NTD is posited away from the C-terminal domain [49]. Complementary hydrogen-deuterium exchange mass spectrometry analysis (HDX-MS) enabled these investigators to further delineate how FIP200 serves to scaffold the assembly of other ULK complex components [49]. 

## 3. Structural Investigations of the Phosphatidylinositol 3-Kinase Complex

**Figure 2:**
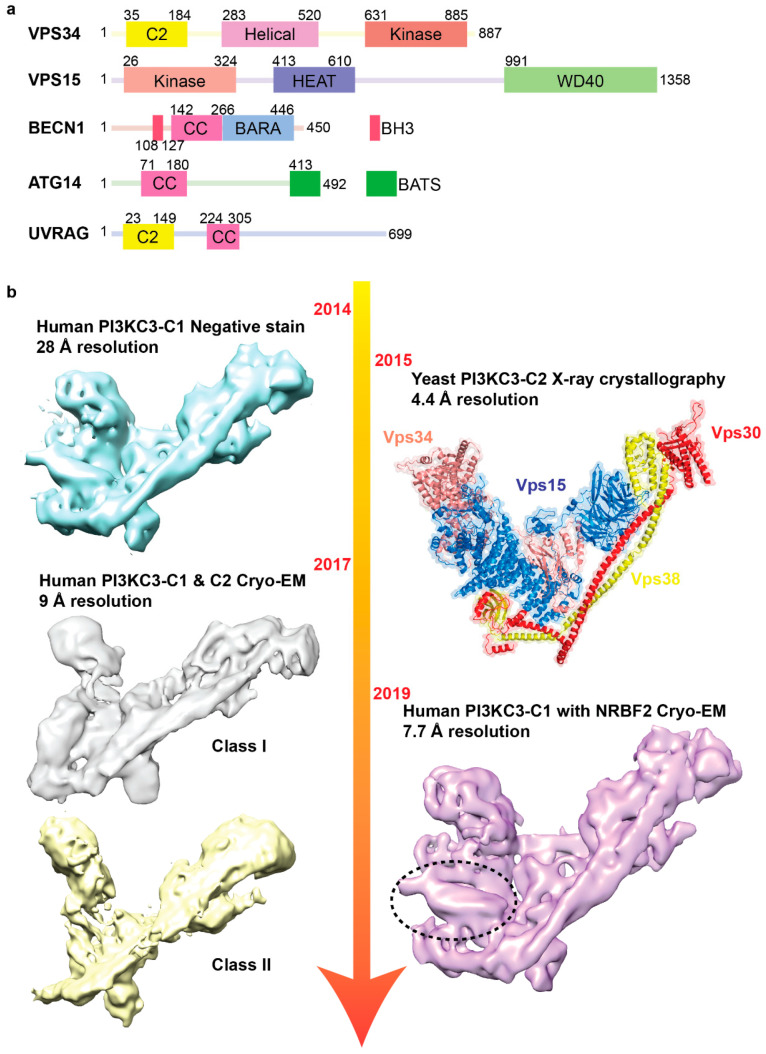
Structural investigations of PI3KC3-C1 and PI3KC3-C2 complexes. (**a**) Schematic diagram of the domain composition of PI3KC3-C1 and PI3KC3-C2 subunits. (CC: coiled coil; BARA: β-α repeat autophagy-specific; BH3: Bcl2-homology 3; BATS: Barkor/ATG14 autophagosome targeting sequence) (**b**) Timeline of PI3KC3 electron microscopy and crystallography structure discovery. From left top to bottom, negative stain EM PI3KC3-C1 structure (cyan) (EMD-2846), cryo-EM structure of PI3KC3-C1 (grey) (EMD-6785) and PI3KC3-C2 (yellow) (EMD-6787). Crystal structure of yeast PI3KC3-C2 (PDB: 5DFZ) shown on the right top and cryo-EM human PI3KC3-C1 with NRBF2 (EMD-20387) at the bottom right (purple). Extra density observed for NRBF2 circled in the black dash.

Also crucial to autophagy initiation is the class III phosphatidylinositol 3-kinase complex (PI3KC3), which is recruited and activated promptly after the Atg1/ULK1 complex. In mammalian cells, PI3KC3 localizes to a specialized region of the ER membrane known as the omegasome where a high level of phosphatidylinositol (PI) are present, and phagophore formation occurs [51]. The main function of PI3KC3 is to produce phosphatidylinositol 3-phosphate (PI3P) from PI, which is important for the recruitment of downstream autophagy factors to expand the phagophore membrane to form the autophagosome [52,53]. In yeast and mammals, PI3KC3 is composed of three core components: The catalytic subunit VPS34 (Vps34 in yeast), VPS15 (Vps15 in yeast), BECN1 (Atg6/Vps30 in yeast). A fourth subunit distinguishes PI3KC3 into two complexes: Complex I (PI3KC3-C1), which contains ATG14 (Atg14 in yeast), and complex II (PI3KC3-C2), which contains UVRAG (Vps38 in yeast), respectively. PI3KC3-C1 has been shown to be involved in autophagy initiation, while PI3KC3-C2 serves multiple roles, including autophagosome maturation, endosomal fusion, Golgi-ER retrograde transport, and endocytic trafficking [54,55,56,57,58]. The catalytic VPS34 subunit, which contains a lipid-binding C2 domain and a helical domain, has lipid kinase activity [59,60,61]. VPS15 regulates VPS34 activity and contains HEAT and WD40 repeats, as well as a putative protein kinase domain [62,63]. BECN1 has an intrinsically disordered region, a BH3 (Bcl2-homology 3, Bcl-2 binding site) domain, a coiled-coil, and a BARA (β-α repeat autophagy-specific) domain [64,65]. ATG14, also known as ATG14L or BARKOR, which recruits the PI3KC3 complex to the ER, contains an N-terminal cysteine repeat region for ER localization, a coiled-coil region, and a C-terminal BATS (Barkor/ATG14 autophagosome targeting sequence) domain [66,67]. UVRAG is involved in membrane trafficking in different cellular pathways and contains an N-terminal MIT (microtubule interacting and trafficking) domain, a C2 domain, and a coiled-coil domain [68] (Figure 2a). 

While X-ray crystallography was successfully used to obtain structural information of the yeast PI3KC3 subunit and their domains, including the coiled-coil region of BECN1 (PDB: 3Q8T) [69], the WD40 domain of Vps15 (PDB: 3GRE) [70], and the catalytic domain of Vsp34 (PDB: 4PH4) [71], until recently little was known about the overall architecture and subunit organization of the fully assembled PI3KC3. The Hurley and Nogales groups made a breakthrough by using negative stain EM to visualize human PI3KC3-C1 and PI3KC3-C2 complex for the first time [72]. 2D analysis revealed that the two related complexes adopt similar overall architecture with an overall V-shape featuring two arms that are approximately 20 nm in length. 3D reconstruction of PI3KC3-C1, along with negative stain EM-based maltose-binding protein (MBP) labeling analysis of each subunit, generated insights into the subunit arrangement of this complex and fits the atomic homology models of the VPS34, VPS15, and BECN1 domains into the EM density map (Figure 2b) [72]. The subunit arrangement and overall architecture of the PI3KC3 deduced from the low-resolution negative stain EM data were subsequently validated by the 4.4 Å crystal structure of yeast PI3KC3-C2 that was determined by the Williams group in the following year [73]. Importantly, this crystal structure provided details on the secondary structures of each subunit that generated the basis for gaining further structural insights into these related complexes and their regulatory mechanisms using negative stain EM and cryo-EM. 

The main challenge in attaining higher resolution in cryo-EM analysis of the PI3KC3′s is their inherent conformation flexibility. The Wang group overcame some of these difficulties and succeeded in obtaining the first cryo-EM maps for both human PI3KC3-C1 and PI3KC3-C2 [74]. Their ~9Å cryo-EM reconstructions confirmed that these two related complexes adopt an overall V-shaped architecture with one arm slightly more elongated than the other. Using their EM density maps and the yeast PI3KC3-C2 crystal structure, these investigators generated pseudoatomic models for both complexes, and these models revealed common features shared between these assemblies. Notably, BECN1 heterodimerizes with ATG14 (PI3KC3-C1) or UVRAG (PI3KC3-C2) through an interwound helical bundle along one “arm” with the N-termini of these proteins pointing towards the base of the V-shape and their C-termini extending to the tip of this arm. The VPS15 WD40 domain contacts the middle region of BECN1 and the coiled-coil region of ATG14 or UVRAG, while its HEAT repeat domain and kinase domain bind the β-barrel of the N-terminal UVRAG and the kinase domain of VPS34, respectively [72,73,74]. The C-terminal domain of VPS34 was found to be conformationally flexible. PI3KC3 has been previously shown to sense and interact with membranes. These investigators applied a novel lipid monolayer-based method that enabled them to examine the orientation of PI3KC3-C1 on the membrane by negative stain EM. Their results showed that PI3KC3-C1 orients in such a way that the tips of the arms containing the C-terminal BATS domain of ATG14 and the CTD of VPS34 contact the lipid monolayer. 

A puzzling question that arises from these structural data is how the regulatory BECN1 and ATG14 subunits modulate the activity of the VPS34 catalytic domain as they do not make direct contact with this domain. To address this question, the Hurley group used negative stain EM to more systematically analyze the conformational dynamics of human PI3KC3-C1. They were able to identify five distinct conformational states that showed different conformations of the VPS34 catalytic domain relative to the core complex [75]. Together with data obtained by chemically cross-linking coupled to mass spectrometry, these investigators discovered that the VPS15 subunit, which was thought to primarily serve as a scaffold for PI3KC3-C1 assembly, plays a role in constraining the movement of VPS34. Another question that arises was how a putative fifth subunit of PI3KC3-C1, known as Atg38 in yeast and NRBF2 in mammalian cells, enhances the activity of this complex. Initial negative stain-based EM labeling studies in conjunction with HDX-MS by the Hurley group revealed that NRBF2 localizes to the base of PI3KC3-C1 [76]. In a follow-up study, these investigators generated a non-dissociable NRBF2-bound PI3KC3-C1 by fusing the MIT domain of NRBF2 to BECN1 [77]. Unexpectedly, this strategy constrained the overall conformational flexibility of PI3KC3-C1 and enabled them to use cryo-EM to better resolve the structural features of this complex. The initial ~7.7 Å resolution 3D reconstruction that they obtained for the NRBF2-BECN1 fusion complex allowed docking of a high-resolution structural model of NRBF2 into the EM density map. A subsequent ~6.6 Å resolution reconstruction obtained for the fusion complex incubated with full-length NRBF2 allowed more precise visualization of the VPS34 kinase domain. When comparing their structure representing the active state of PI3KC3-C1 to the previously reported crystal structure of yeast PI3KC3-C2 representing the inactive state, these investigators were able to identify the movement that subunits make as the complex transition from the inhibited state to the active state. 

Building on this foundation, these investigators next focused on understanding the mechanism of action, a negative regulator of autophagy known as Rubicon. Rubicon has been shown to bind tightly to PI3KC3-C2 and inhibits its activity [55,78]. These investigators first used a combination of HDX-MS and pulldown assays to identify the minimal binding region termed PIKBD [79]. Using a similar strategy as NRBF2, they engineered a PI3KC3-C2 complex more amenable to cryo-EM by fusing Rubicon PIKBD to the C-terminus of BECN1 to prevent dissociation of Rubicon. In addition, they incorporated into this re-engineered PI3KC3-C2 a VPS34-VPS15 fusion protein that, based on their previous work, would stabilize the V-shape conformation. With this modified complex, they were able to obtain a ~6.8 Å cryo-EM reconstruction, which allowed them to visualize how Rubicon PIKBD contacts the BARA domain of BECN1 and potentially obstructs its interaction with the membrane. 

## 4. Structural Investigations of Atg9

Atg9 (ATG9A and ATG9B in mammalian cells) is the only transmembrane protein of the core autophagy machinery. Atg9 localizes to the trans-Golgi network (TGN) and late endosome, as well as traffics freely in between these compartments in single-membrane cytoplasmic vesicles termed Atg9 vesicles [35,80]. With a diameter of 30 to 60 nm, these Atg9 vesicles are crucial to autophagosome biogenesis by providing a source of membrane for phagophore nucleation at the PAS in yeast cells [35,36,81]. In yeast, Atg9 vesicles are recruited to the PAS and tethered by either Atg11 during selective autophagy or Atg17 during non-selective autophagy. The tethered Atg9 vesicles integrate into the outer membrane of the phagophore and cluster at the extremities of the growing phagophore [82,83,84]. On the other hand, ATG9 in plants and mammals do not reside on the phagophore, but rather associate transiently with the phagophore and are recycled back to TGN and the cytoplasmic pool [85,86]. Although Atg9 vesicles provide a source of membrane for phagophore formation, addition membrane required for phagophore expansion and maturation into autophagosome is provided by the Atg2-Atg18 complex, which transfers lipids from the ER and the omegasome to the growing phagophore. Studies have shown that Atg2 binds to Atg9 and Atg1-phosphorylated Atg9 recruits Atg18 to form an Atg9-Atg2-Atg18 complex [84,87]. However, the exact role of Atg9 in this complex remained unclear. 

Structural studies of Atg9 have been hampered by challenges in producing a sufficient amount of stable membrane proteins. Lau and colleagues developed a procedure to produce recombinant *Arabidopsis thaliana* ATG9 (AtATG9) solubilized in the detergent LMNG, and this enabled them to use single-particle cryo-EM to obtain the first structural data on this membrane protein [88]. Their initial 2D analysis revealed that the purified sample is composed of a mixture of monomeric, dimeric, and trimeric AtATG9. These investigators focused their subsequent analysis on the trimeric form and, through using different image processing tricks, was able to obtain a map of ~7.8Å. Although the limited resolution precluded the construction of an atomic model, the map enabled them to deduce that each protomer contains at least six transmembrane segments, with two of them highly tilted. Through map segmentation, these investigators were able to assign volumes constituting the cytoplasmic N-terminal and C-terminal regions. Based on their putative structural model, AtATG9 would oligomerize via both the cytoplasmic region and transmembrane region [88]. 

**Figure 3:**
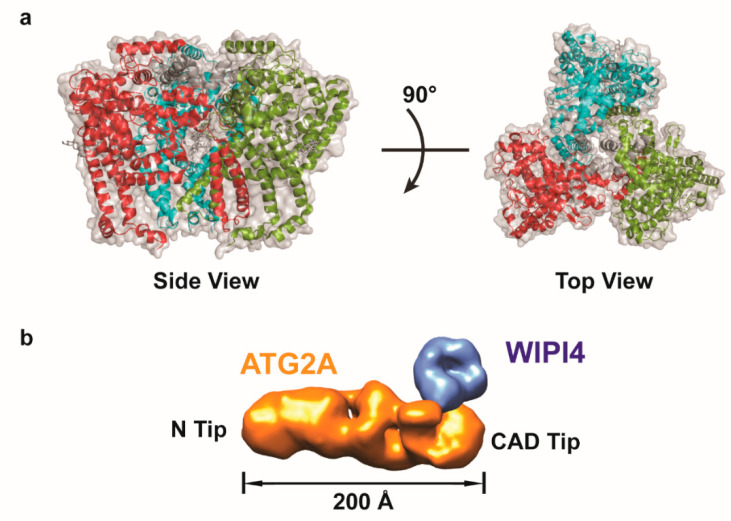
3D EM reconstructions of ATG9A and ATG2A-WIPI4 complex. (**a**) Side view and top view of the cryo-EM reconstruction of the trimeric human ATG9A (state A) (PDB: 6WQZ). The three ATG9A monomers are shown in the red, green, and cyan cartoon, respectively. (**b**) Negative stain EM reconstruction of the human ATG2A-WIPI4 complex (EMD-8899). The complex shows a club-shaped architecture, with a ~200 Å rod-shaped ATG2A (in gold color) and WIPI4 (in blue color) bound to the CAD tip of the rod. The N-tip is located on the opposite end of the CAD tip.

Very recently, the Banerjee group made a major breakthrough by determining the high-resolution cryo-EM structure of the human ATG9A (Figure 3a) [89]. This was enabled by developing a more robust procedure to produce highly purified and homogeneous trimeric human ATG9A solubilized in the detergent LMNG. At a resolution of 2.9 Å, the cryo-EM map obtained by them, allowed de novo building of an atomic model that shows a fold unique to other previously characterized membrane proteins. More specifically, their structural model indicated that human ATG9A consists of three triangular wedge-shaped protomers, with each protomer composed of 22 alpha helices and four beta strands. Contrary to what was proposed for AtATG9 [88], each ATG9A protomer consists of four transmembrane helices and two helices that are partially embedded in the membrane [89]. Extensive domain swapping was observed within the ATG9A trimer, where two helices from one protomer traverse and interact with another two helices from the adjacent protomer to form the transmembrane domain, thus facilitating self-association [89]. The ATG9A atomic model revealed a branched network of cavities consisting of a central pore, a lateral branch that continues from the central pore to the membrane cytosolic border, and a perpendicular branch that extends from the lateral branch to the cytoplasm. Interestingly, these cavities are lined with hydrophilic residues that might function to transport phospholipids through interaction with their polar head groups. Blocking the central pore and lateral branch by introducing a bulky tryptophan residue in each cavity resulted in the formation of enlarged, aberrant autophagosomes, suggesting that the transport function of the cavity is important for autophagy [89]. Subsequently, systematic C-terminal truncation mutagenesis, in conjunction with protein-protein interaction assays, enabled localization of the binding site for ATG2A, where the cytosolic C-terminal helical platform is important for ATG9A-ATG2 interaction [89]. Altogether, the authors postulated that the ATG9A-ATG2 complex serves to transport lipids from ATG2 to ATG9A to the growing phagophore. 

## 5. Structural Investigations of the Atg2-Atg18 Complex

With an overall mass of ~200kDa, Atg2 (ATG2A and ATG2B in mammalian cells) is a large-sized and highly conserved component of the core autophagy machinery. Atg2 contains a Chorein_N domain that shares sequence similarity with the N-terminus of the Vps13 family proteins that function to mediate lipid transport between organelles [90]. Atg2 forms a complex with the 7-bladed β-propeller protein Atg18 (WIPI1, WIPI2, WIPI3/WDR45B, and WIPI4/WDR45 in mammalian cells) that binds PI3P and acts as a downstream effector [91,92,93,94]. The Atg2-Atg18 complex localizes to the edge of the growing phagophore and facilitates the phagophore-ER/omegasome association [84,95]. The Atg2-Atg18 is essential for autophagosome formation as their deficiency resulted in unclosed autophagosome and disruptive autophagic activity. 

Until very recently, the only structural information available for Atg2-Atg18 has been limited to high-resolution structures of the yeast Atg18 homolog HSv2 and human WIPI3 obtained by X-ray crystallography [96,97,98]. The Yu and Wang groups first applied negative stain EM in an attempt to gain knowledge on the overall architecture of the Atg2-Atg18 complex [99]. Their pioneering work resulted in a ~18 Å 3D reconstruction of the mammalian ATG2B-WDR45 complex, showing that this complex has an overall club-shaped architecture composed of a ~22 nm-long rod-shaped ATG2B and a small globular-shaped WDR45 attached at one end of the rod. Complementary negative stain EM-based labeling analysis revealed that the ATG2B N-terminus is located at the end of the rod opposite to the WDR45 bound tip. Subsequent negative stain EM studies by the Otomo group on the human ATG2A-WIPI4 complex and the yeast Atg2-Atg18 complex not only confirmed these findings, but also showed that the overall architecture of this complex is conserved from yeast to human (Figure 3b) [100]. Complementary chemically cross-linking coupled to mass spectrometry analysis data obtained by these investigators further revealed that ATG2A is folded into a non-linear chain topology, while ATG2A is cross-linked to blade 2 and blade 3 on WIPI4, consistent with the previous findings where the yeast Atg2 interacts with blade 2 on Atg18 [101]. Further site-by-site mutagenesis analysis revealed that the Y/HF motif within the C-terminus of ATG2A/B is crucial for WIPI binding. MBP tagging approach was also employed in this study, where fusing the MBP tag to the CAD domain, a conserved domain located in the middle of ATG2A’s sequence, resulting in an additional density located at the WIPI4 binding-end of the rod (hereafter CAD-tip). Interestingly, both the N-terminus and the CAD-tip were observed to bind high-curvature vesicles based on negative-stain single-particle EM analysis. Collectively, these results pointed to a possible role of ATG2 as a bipartite membrane tethering protein. 

Despite these advances, the new structural data did not provide a clear answer regarding the precise molecular function of the Atg2-Atg18 complex. The breakthrough came when the Noda group determined the crystal structure of the N-terminal region of *S. pombe* Atg2. This structure revealed that Atg2 contains a hydrophobic cavity, and through additional biochemical analysis, this cavity is capable of binding phospholipids. Based on these results, the Noda group proposed that Atg2 serves as a lipid transferring protein [102]. Two subsequent cryo-EM based studies would validate this hypothesis. First of all, the Otomo group examined the human ATG2A-WIPI4 complex by cryo-EM [103]. Although they were unable to obtain a 3D reconstruction, due to sample heterogeneity, the 2D analysis showed an internal cavity connected between the N-tip and the CAD-tip within ATG2A. At around the same time, the Melia/Reinisch/Walz groups succeeded in determining the cryo-EM reconstruction of ATG2A [104]. Although this 3D reconstruction was only at ~15 Å, it showed a cavity that extends from the N-tip to the CAD-tip. With a diameter of 20 Å, this cavity is sufficiently large to accommodate multiple glycerophospholipids concurrently. These structural observations, along with results from in vitro lipid transfer assays, led to the proposal that ATG2 serves the important function of transferring lipids from the ER to support the growth of the phagophore during autophagosome biogenesis. 

## 6. Structural Investigations of Selective Autophagy Components

**Figure 4:**
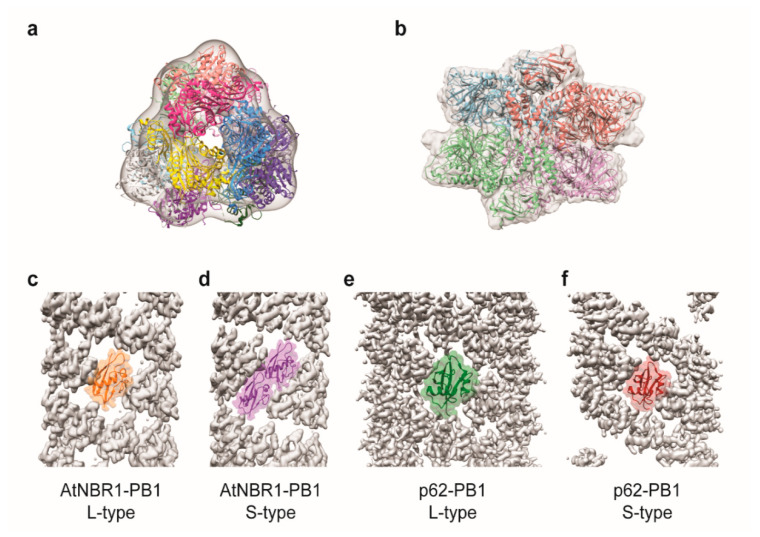
3D EM reconstructions of selective autophagy components. (**a**) Crystal structure of *S. cerevisiae* mApe1 (PDB: 5JM9) fitted into the cryo-EM map of *S. cerevisiae* mApe1 dodecamer (EMD-8167). Each chain is shown in a different color. (**b**) Crystal structure of *S. cerevisiae* Ams1 (PDB: 5JM0) fitted into a cryo-EM map of *S. cerevisiae* Ams1 tetramer (EMD-8166). Each chain is shown in a different color. (**c**–**f**) Cryo-EM reconstruction of *Arabidopsis thaliana* NBR1 (AtNBR1) PB1 domain and p62-PB1 domain filaments, with each of the crystal structure fitted into the cryo-EM map of the filament subunit. (c) AtBNR1-PB1 monomer (in orange) (PDB: 6TGN) in AtBNR1-PB1 L-type filament (EMD-10499). (d) AtBNR1-PB1 homodimer (in purple) (PDB: 6TGP) in AtBNR1-PB1 S-type filament (EMD-10500). (e) p62-PB1 monomer (in green) (PDB: 6TGY) in p62-PB1 L-type filament (EMD-10501). (f) p62-PB1 monomer (in red) (PDB: 6^TH^3) in p62-PB1 S-type filament (EMD-10502).

A key model for dissecting the basic principles of selective autophagy is the yeast *S. cerevisiae*-specific cytoplasm-to-vacuole targeting (Cvt) pathway. This pathway is considered a type of selective autophagy as it uses the core autophagy machinery, as well as specialized adaptor proteins to sequester hydrolytic enzymes into double-membrane autophagosome-like transport vesicles known as Cvt vesicles [13,105,106]. The Cvt vesicles are targeted to the yeast vacuole, where these enzymes mature and degrade materials inside the lumen of the vacuole [107,108,109,110]. The major cargo of the Cvt pathway is aminopeptidase I (Ape1), which is synthesized as a zymogen containing an N-terminal pro-peptide (prApe1) in the cytoplasm and subsequently forms a homododecamer. The pro-peptide then mediates the formation of an aggregate known as the Ape1 complex [111,112,113]. The Ape1 complex is recognized by the selective receptor Atg19, which links this cargo to the core machinery for encapsulation. Single-particle EM has contributed to understanding the structural features of prApe1, which is aggregation-prone and technically challenging to work with. Notably, Chang and colleagues used negative stain EM to obtain the first structural information on prApe1—a 3D reconstruction of detergent solubilized *S. cerevisiae* prApe1 [114]. This EM structure revealed the overall tetrahedral architecture of homododecameric prApe1 and enabled them to propose a subunit organization model for this oligomer. A subsequent negative stain EM investigation by the Sachse group on non-detergent solubilized *S. cerevisiae* prApe1 revealed the propensity of prApe1 to form higher-order oligomers compared to mature Ape1 lacking the pro-peptide (mApe1) (Figure 4a) [115]. These investigators also examined how Atg19 interacted with prApe1 by negative stain EM and showed that this adaptor binds to the periphery of the “tetrahedron” [115]. Interestingly, Atg19 binding reduces the ability of prApe1 to form larger-sized oligomers [115]. A secondary cargo of the Cvt pathway is α-mannosidase 1 (Ams1). The Sachse group succeeded in determining a 6.3 Å structure of *S. cerevisiae* Ams1 by cryo-EM (Figure 4b). This cryo-EM structure revealed the unique tetrameric oligomeric state adopted by Ams1 and enabled the construction of a pseudo-atomic model of this Cvt cargo [115]. 

The first selective autophagy adaptor discovered in higher eukaryotes is p62 [116]. Also known as sequestosome 1 (SQSTM1), p62 contains three domains: An N-terminal PB1 domain that mediates homo- and hetero-oligomerization, a C-terminal UBA domain that binds ubiquitinated proteins, and an LC3-interaction region (LIR) [116,117,118,119] that mediates interaction with LC3/ATG8 family of proteins that localize to the autophagosomal membrane. It is thought that this domain arrangement allows p62 to link ubiquitinated proteins and to the growing phagophore for sequestration into the autophagosome [117]. p62 has also been shown to form the so-called p62 bodies in the cytoplasm by clustering misfolded proteins and protein aggregates into a single site. These p62 bodies are thought to serve as a scaffold for initiating autophagosome formation [120]. Single-particle EM has played a pivotal role in informing us about the structural features of p62. Notably, pioneering EM work by the Sachse group led to the discovery that the p62 PB1 domain forms a flexible helical filament with significant curvature and pitch variation. They subsequently determined a ~10 Å structure of this polymer by cryo-EM, and their EM density map provided an envelope for fitting the crystal structure of the PB1 domain, which generated insight into the organization of this domain within this polymer, as well as the importance of a short motif at the C-terminal region of the PB1 domain in stabilizing this assembly [120]. These investigators also demonstrated that full-length p62 form “ribbon-like polymers”, but the irregularity and flexibility of these polymers precluded higher-resolution analysis [120]. Interestingly, when they mixed K63-linked octa-ubiquitin to full-length p62, they observed, by negative stain EM, the shortening of the p62 polymers [121]. This shortening does not occur for p62 lacking the UBA domain, confirming the importance of UBA-ubiquitin interaction in this phenomenon. More recently, the Sachse group expanded their analysis to the other PB1 domain-containing proteins, including the NBR1 autophagy receptor from *A. thaliana* (AtNBR1). Their negative stain analysis revealed that the AtNBR1 PB1 domain also forms filamentous polymers like p62 PB1 [121]. Furthermore, they were able to harness the power of new EM hardware to determine the high-resolution cryo-EM structures of both the AtNBR1-PB1, as well as the p62-PB1 filaments in two different configurations (ladder-like or L-type and serpent-like or S-type) (Figure 4c). These high-quality maps enabled them to construct atomic models of these filaments [121]. These structural models generated insights into the molecular mechanism of polymerization by revealing the subunit organization pattern and the importance of a conserved “double arginine finger” motif that stabilizes the assembly [121]. 

## 7. The Road Ahead

From the humble beginning of characterizing the overall architecture of Atg17-Atg29-Atg31 subassembly of the yeast Atg1 complex to the very recent sub-3Å structure of human ATG9A, single-particle EM has established itself as an indispensable research tool in the autophagy research field in less than a decade. Notably, the ability of single-particle EM to directly visualize individual proteins/protein complexes and the relatively low sample requirement and stringency enabled structural investigations of autophagy regulators that have previously eluded crystallographic and NMR spectroscopic analyses. The exciting data generated from single-particle EM investigations, combined with previous crystal and NMR structures and results from in vitro biochemical and reconstitution studies, have transformed our structural understanding of proteins and protein complexes involved in selective and non-selective autophagy. A recent surge in the establishment of local and national EM facilities has made single-particle EM technology more accessible to the general research community [16,17,122]. We foresee that more autophagy researchers will adopt single-particle EM technology. This, together with further improvement in cryo-EM specimen preparation methods, EM hardware, and image processing algorithms, will usher us into a golden era of structural investigations of the autophagy machinery. In addition to an increase in the number of high-quality, high-resolution structures being reported in the next few years, we will likely witness the visualization of smaller-sized proteins and assemblies (<100 kDa) that are currently at or below the threshold of detection of EM imaging. 

A common theme gathered from previous single-particle EM investigations in the autophagy field is that the majority of the proteins and protein complexes involved in this degradation pathway are conformationally flexible. The development of the Volta phase plate for producing images with enhanced contrast [123,124] and new algorithms, such as multibody refinement and 3D variability analysis that allows more systematically examination of the conformational landscape of imaged particles [125,126] are pushing the boundary of the range of dynamic proteins and protein complexes that are amenable to cryo-EM. Nonetheless, as exemplified by the very recent structural characterization of human FIP200, there are many Atg proteins/proteins that will continue to be very difficult to be analyzed by high-resolution cryo-EM methods. Thus, negative stain single-particle EM, despite its lower resolution and its potential to introduce artifacts, due to sample flattening and drying, will continue to serve an indispensable role for quality control in the pre-cryo-EM phase, as well as the acquisition of 2D and 3D structural data for a number of these proteins and assemblies. In the past few years, we have also witnessed the introduction of other complementary structural approaches and, most notably, structural proteomics techniques, including HDX-MS and cross-linking coupled to mass spectrometry in the autophagy field. These methods yielded important data regarding the secondary structure arrangement of proteins and/or subunit organization of different dynamic assemblies. These techniques, as well as X-ray crystallography and NMR spectroscopy, will continue to be relied on as analyzing dynamic properties of proteins and protein complexes involved in autophagy necessitate the use of integrated structural approaches. 

A key strength of single-particle EM is that it images proteins and protein complexes in their solution state as opposed to in an ordered lattice as in X-ray crystallography. However, whether the biochemical and structural features that we observe in vitro truly represent how these proteins behave and function within the context of the physiological environment inside cells remain unknown in most cases. Another common property observed for many components of the autophagy machinery is their propensity to interact and engage with cellular membranes. A potential strategy to bridge the gap between in vitro and in vivo studies would be to capture structural information of these proteins and protein complexes bound to membranes. The lipid monolayer approach employed by the Wang group in determining the orientation of PI3KC3 on the membrane [74] could be potentially employed in studying other components of the autophagy machinery. On the other hand, established membrane mimetics, such as liposomes and nanodiscs, offer alternative approaches that might enable high-resolution cryo-EM analysis. Ultimately, to truly address this “resolution gap” problem would require us to visualize these proteins and protein complexes in their native environment inside a living cell. One technique that can tackle this problem is correlative light and electron microscopy (CLEM). CLEM, which images the same cellular specimen sequentially using a fluorescence microscope and a transmission electron microscope, combines the strength of fluorescence microscopy in localizing specific cellular and subcellular target and the strength of transmission electron microscopy in capturing high-resolution details of subcellular structures [127,128]. The power of CLEM was vividly demonstrated in a very recent study by the Sachse group, which used CLEM to characterize the ultrastructural details of p62 bodies in mammalian cells [121]. Intriguingly, their work revealed a meshwork of filaments reminiscent of those observed in the cryo-EM analysis of p62 filaments at these p62 bodies. A technical challenge in EM imaging of cells is that most eukaryotic cells are too thick to be directly imaged with a transmission electron microscope. An approach that can overcome this challenge is cryo-focused ion beam milling or cryoFIB, which uses a gallium ion beam to generate thin lamellae specimens from vitrified cell samples as cryo-focused ion beam milling [129,130,131,132,133]. The recent improvement in instrumentation and experimental workflow have dramatically improved the success and throughput in producing high-quality thin specimens [134,135,136]. Specimens obtained from cryoFIB can then be transferred to a transmission electron microscope to be examined by cryo-electron tomography (cryo-ET), which involves acquiring a series of images of the specimens at different tilt angles. These images can be used to reconstruct a 3D tomogram that allows direct visualization of proteins and protein complexes in their cellular context. 

In spite of the exciting progress in the structural elucidation of the autophagy machinery, a growing body of experimental data has suggested that phase separation is an important part of the autophagy degradation process. For example, a landmark study from the Noda group demonstrated that the yeast Atg1 complex undergoes phase separation critical to forming the phagophore assembly site [137]. Although studying the properties and dynamics of these liquid-like condensates and their biogenesis is beyond the capability of conventional structural techniques, including single-particle EM, structural information is still crucial to facilitate a better understanding of the molecular basis of biogenesis and morphogenesis of these unique entities within the cell. 

## Abbreviations

2D	two-dimensional
3D	three-dimensional
Ams1	α-mannosidase 1
Ape1	Aminopeptidase I
ATG	Autophagy-related
BARA	β-α repeat autophagy-specific
BARKOR	Beclin 1-associated autophagy-related key regulator
BATS	Barkor/ATG14 autophagosome targeting sequence
BECN1	Beclin1
BH3	Bcl2-homology 3
CLEM	Correlative light and electron microscopy
cryo-ET	cryo-electron tomography
cryoFIB	cryo-focused ion beam
CTD	C-terminal domain
Cvt	Cytoplasm-to-vacuole targeting
EM	Electron microscopy
ER	Endoplasmic reticulum
FIP200	FAK family kinase-interacting protein of 200 kDa
HDX-MS	Hydrogen-deuterium exchange mass spectrometry
HEAT	Huntingtin, elongation factor3 (EF3), protein phosphatase 2A (PP2A), TOR1
HORMA	Hop1, Rev7, and Mad2
LMNG	lauryl maltose neopentyl glycol
mApe1	Mature Ape1
MIT	Microtubule interacting and trafficking
NBR1	Next to BRCA1 gene 1
NMR	Nuclear magnetic resonance
NRBF2	Nuclear receptor binding factor 2
NTD	N-terminal domain
PAS	Phagophore assembly site
PI	phosphatidylinositol
PI3KC3	class III phosphatidylinositol 3-kinase
PI3KC3-C1/2	class III phosphatidylinositol 3-kinase complex I/II
PI3P	phosphatidylinositol 3-phosphate
PIKBD	phosphatidylinositol 3-kinase binding domain
prApe1	precursor aminopeptidase I
PB1	Phox and Bem1
SQSTM1	Sequestosome-1
TGN	trans-Golgi network
TOR	target of rapamycin
TORC1	target of rapamycin complex 1
ULK	Unc-51 like autophagy activating kinase
UVRAG	Ultraviolet irradiation resistance-associated gene
VPS	Vacuolar protein sorting
WDR45/46	WD repeat domain 45/46
WIPI	WD-repeat protein interacting with phosphoinositides
